# Activity induces traveling waves, vortices and spatiotemporal chaos in a model actomyosin layer

**DOI:** 10.1038/srep20838

**Published:** 2016-02-15

**Authors:** Rajesh Ramaswamy, Frank Jülicher

**Affiliations:** 1 Max Planck Institute for the Physics of Complex Systems (MPI-PKS), Nöthnitzer Str. 38, 01187 Dresden, Germany

## Abstract

Inspired by the actomyosin cortex in biological cells, we investigate the spatiotemporal dynamics of a model describing a contractile active polar fluid sandwiched between two external media. The external media impose frictional forces at the interface with the active fluid. The fluid is driven by a spatially-homogeneous activity measuring the strength of the active stress that is generated by processes consuming a chemical fuel. We observe that as the activity is increased over two orders of magnitude the active polar fluid first shows spontaneous flow transition followed by transition to oscillatory dynamics with traveling waves and traveling vortices in the flow field. In the flow-tumbling regime, the active polar fluid also shows transition to spatiotemporal chaos at sufficiently large activities. These results demonstrate that level of activity alone can be used to tune the operating point of actomyosin layers with qualitatively different spatiotemporal dynamics.

Biological systems like cytoskeletal filaments^1^,^2^,^3^, bacterial suspensions^4^, cell aggregates and tissues^4^, and flocks of birds^5^ are examples of active living matter^2^,^4^,^6^. Such active systems consist of a set of interacting agents that exhibit coordinated motion or flows induced by energy consumption^4^,^6^. Energy consumption in active matter leads to chaotic motion in bacterial suspensions^4^, cell polarity inducing flows in the actomyosin cortex of single cell *C. elegans* embryos^7^,^8^,^9^, and traveling waves and swirling motion of actin filaments *in vitro*^10^. Characterizing and understanding the behavior of active matter is crucial to understand the physics of such biological phenomena and other mechanochemical processes mediating morphogenesis^9^.

Several morphogenetic processes in biological systems are brought about by the actomyosin cortex^11^. The dynamics of the actomyosin cortex play a crucial role in cytokinesis^12^, cell migration^2^,^11^, gastrulation in *Drosophila*^13^, and cell polarity establishment in *C. elegans*^7^,^8^ that are fundamental morphogenetic processes during organism development. The actomyosin cortex is composed of polar actin filaments crosslinked by motor proteins, such as myosin, that undergo conformational changes driven by a chemical fuel. Large numbers of driven conformation changes of motor proteins induce contractile active stresses in the cortex^11^,^14^. The resulting activity that quantifies the contractile active stresses subsequently induce flows in the system making it highly dynamic. Studying activity induced dynamics of active gels such as the actomyosin cortex is therefore essential to understanding morphogenesis^15^.

Inspired by dynamics of cytoskeletal systems such as the actomyosin cortex, the continuum theory of active polar fluids was developed^1^,^2^,^3^,^16^. The theory models the mechanics of uniaxial active agents such as actin filaments, embedded in a viscous bulk medium, in which active stresses are induced due to dissipation of energy^4^,^6^. The average orientation of the agents is characterized by a polarity field. The spatiotemporal dynamics of the polarity field is governed by an equation of motion accounting for energy consumption, alignment or tumbling of the polarity field by local shear flow, and the tendency of the polarity field to resist spatial distortions^2^,^17^. The relationship between the strain rate and the stress in the fluid is provided by a constitutive equation that accounts for polarity and consumption of energy. These equations, along with conservation of momentum, provide a continuum hydrodynamic description of active polar fluids^1^,^2^,^3^,^4^,^16^ characterized by material constants, and a scalar field called activity measuring the active stresses in the system. The nonlinearities in the hydrodynamic equations, however, render the prediction of complex spatiotemporal dynamics analytically intractable.

Earlier studies have revealed interesting dynamics of active polar and nematic fluids. By using linearized hydrodynamical equations, instabilities of spatially-homogeneous steady states have been deciphered^2^,^17^,^18^,^19^,^20^. These studies have predicted spontaneous flow transitions^2^,^18^,^19^, and transitions between polar patterns such as asters, spirals and vortices as a function of activity^2^,^20^. Such transitions have been observed experimentally in the organization of microtubules *in vitro* upon varying the concentration of motor proteins^21^. Numerical approaches have confirmed spontaneous flow transitions^22^,^23^ and transitions between polar patterns^24^ in active polar fluids. Numerical studies have also been used to find a rich variety of patterns in active nematic and polar fluids^25^,^26^,^27^. Additionally, using an extended Toner-Tu model of active fluids, irregular dynamics that could correspond to chaos and possibly turbulence as experientially seen in bacterial suspensions have been observed^28^,^29^. Chaos-like irregular dynamics have also been demonstrated in two-dimensional active nematic and polar fluids where the activity is coupled to the filament concentration governed by an advection-diffusion equation^26^,^27^.

Here, we consider a layer of active polar fluid with finite thickness sandwiched between two plates. At the boundary of the fluid, frictional forces are imposed relative to the surface of the plate. Such a set-up represents a simple model for a layer of active fluid such as the actomyosin cortex that is sandwiched between the cell membrane and the cytosol. The nonlinear dynamics of such an active polar fluid at low Reynolds number subjected to strong spatially-homogeneous activity, however, remains unexplored. We numerically explore the spatiotemporal dynamics as a function of spatially-homogeneous activity of the system. We make use of a recently developed hybrid particle-mesh method to numerically solve the hydrodynamic equations of active polar fluids^24^. The numerical results show that the nonlinear dynamics as a function of activity depend on the nature of interaction between the polarity field and the local shear generated by the flow. In the flow-aligning regime, where the filaments tend to align along the flow direction, we find two transitions as the activity is increased: transition to spontaneous flow, and a transition to traveling waves accompanied by traveling vortices in the flow field. In the flow-tumbling regime, we find an additional transition to spatiotemporal chaos. We characterize this chaotic state by computing the maximum Lyapunov exponent of the spatiotemporal dynamics. The transitions to traveling waves and spatiotemporal chaos are effects that are due to nonlinearities in the hydrodynamics of active polar fluids. This is the first time such transitions have been shown in active polar fluids subjected to spatially homogeneous activity. The results therefore demonstrate that the level of activity alone can tune the operating point of an actomyosin layer characterized by qualitatively different spatiotemporal dynamics.

## Model

**Figure 1:**
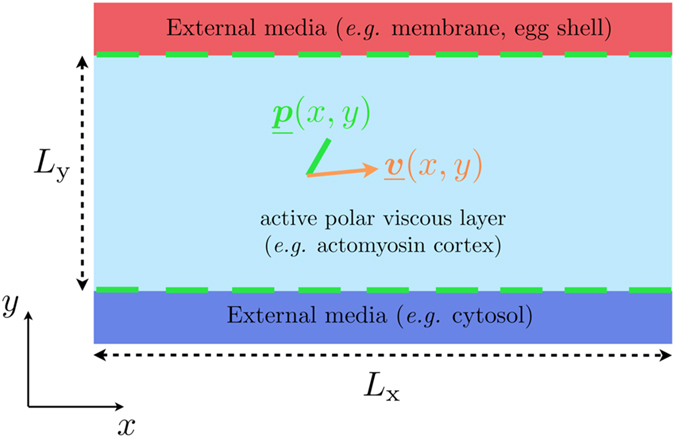
Model illustration. Model active polar fluid layer sandwiched between two external media. The sandwiched model active layer spans a length of 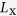 in the x-direction and 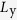 in the y-direction. The layer is sandwiched between *y* = 0 and 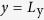.

We consider a two-dimensional active polar fluid in the x-y plane described by a continuum hydrodynamic theory (see Hydrodynamic equations of active polar fluids in Sec. Methods). This corresponds to the case of a three-dimensional system with translational invariance and zero polarity component in the z-direction. The x and y components of the polarity field 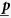 at each point is denoted by 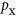 and 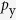, such that 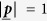. The components of the velocity field 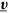 are denoted by 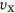 and 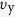. The fluid has a thickness 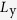 in the y-direction, and length 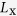 in the x-direction. We impose a friction boundary condition for the flow along 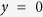 and 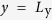 so that the shear stress 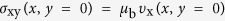 and 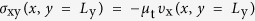, where 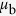 and 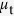 denote the friction coefficients at the bottom and top surfaces respectively. This flow boundary condition is a generalized slip boundary condition that models the effect of (different) frictions due to the cytosol on one side of the actomyosin gel and the membrane on the other. The normal component of the velocity 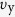 at 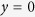 and 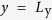 vanishes. The polarity along the surface 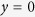 and 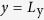 are anchored parallel to the surface (see Fig. 1 for an illustration of the model).

The hydrodynamic model is parametrized by the following material constants: viscosity of the fluid 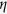, orientational friction of the polar filaments *γ*, the elastic constants of the polarity field *K* (considering 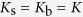), and a coefficient 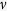 coupling the rate of change of polarity with the strain rate (see Hydrodynamic equations of active polar fluids in Sec. Methods for more details). The fluid is subjected to activity 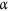 that is spatially-homogeneous. We choose these parameters by constraining our active polar fluid model to be contractile^11^,^14^ and spontaneously flowing like an actomyosin cortex in biological cells^7^,^8^. First, in order to ensure that the active stresses are contractile, we enforce that 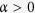 (see Eq. 1). In addition, spontaneous flow of the fluid requires that the coupling coefficient 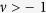 (see Sections Critical activity α_c_ for spontaneous flow transition and Model parameters in Sec. Methods for details). If 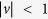, the active fluid model is known to be in a flow-tumbling regime and in the flow-aligning regime otherwise^17^,^22^. Individual actin filaments have been observed to be flow-tumbling^30^ supported by theoretical predictions and experimental observations of similar rod-like polymeric liquid crystals^31^. Response of individual actin filaments that are chemically interacting with other biomolecules in an *in vivo* actomyosin cortex is, however, unclear. We therefore study the flow patterns in the flow-tumbling as well in the flow-aligning regimes. See Model Parameters in Sec. Methods for the values of all parameters for the two regimes used in our study.

With the given parametrization of the model and periodic boundary condition in the x-direction, we numerically solve the equations governing the hydrodynamics using a recently developed general hybrid particle-mesh method for incompressible active polar viscous gels^24^. The method imposes the unit vector polarity and incompressibility constraints exactly, and has shown to be consistent, stable and therefore convergent^24^. The initial condition and settings used for the numerical simulations are presented in Sec. Methods (see Settings for numerical simulation).

## Results

We present the numerical solution to the hydrodynamic equation governing the model system^24^. We study the polarity and flow dynamics as activity 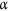 induces larger contractile stresses in the active polar fluid model. Specifically, we characterize the polarity and flow dynamics as 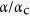 is increased over two orders of magnitude where 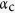 is the critical activity (lower bound of Eq. 9) beyond which spontaneous flows occur. Over this range of activities, nonlinearities in the hydrodynamics become significant and cannot be ignored. As the activity is increased, we observe that the contractile active polar fluid in the flow-tumbling regime undergoes 3 transitions: spontaneous flow transition, transition to oscillatory dynamics, and finally a transition to spatiotemporal chaos. In contrast, the flow-aligning regime only shows 2 transitions: the spontaneous flow transition and the transition to oscillatory dynamics. The oscillatory dynamics and the chaotic dynamics are not predicted by the linearized equations. These transitions are therefore due to the nonlinearities in the hydrodynamic equations that are neglected in the linearized regime. In the following sections, we present the results in the flow-tumbling regime before summarizing the differences for the flow-aligning regime towards the end of the section.

### Spontaneous flow

**Figure 2:**
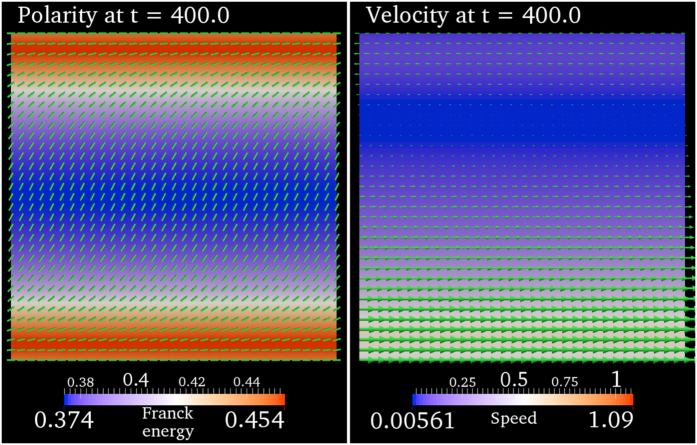
Spontaneous flow. Steady-state polarity and velocity fields for (*α*/*α*_c_) = 3 after a long time *t* = 400. The polarity field is indicated by cylindrical rods. The Franck free-energy density of the polarity field (Eq. 5) is color coded. In the velocity plot, the arrows denote local velocity normalized by the maximum magnitude of velocity across the computational domain. The direction of the arrows therefore indicate the local flow direction, and the length of the arrows indicates the relative magnitude of velocity. The local speed of flow is color coded. See Sec. Model for model details and Sec. Model Parameters in Sec. Methods for the parameters used to simulate the model. The horizontal direction towards the right is the positive x-direction and the vertical direction towards the top is the positive y-direction.

For activities such that 0 < (*α*/*α*_c_) < 1, the steady-state polarity field is spatial homogeneous and the velocity is zero. When 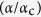 is increased beyond 1, the active polar fluid undergoes a spontaneous flow transition as predicted by the linear perturbation analysis (see Critical activity *α*_c_ for spontaneous flow transition in Sec. Methods). Figure 2 shows the steady-state polarity and velocity fields when (*α*/*α*_c_) = 3 for the flow-tumbling regime. We observe that the steady-state polarity and velocity fields are translationally invariant along the x-direction with a finite velocity in the x-direction. The translational invariance in the *x*-direction along with the incompressibility constraint render velocity 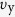 in the y-direction zero. The velocity 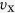 along the x-direction, however, is finite owing to non-zero gradients in the polarity field in the y-direction.

In summary, the numerical solution confirms the theoretical prediction of spontaneous flow transition beyond critical activity. Previous numerical studies have also confirmed such spontaneous flow transition in active polar fluids albeit with no slip boundaries^22^,^27^ instead of the friction boundary conditions used in the current study.

### Traveling waves and traveling vortices

**Figure 3:**
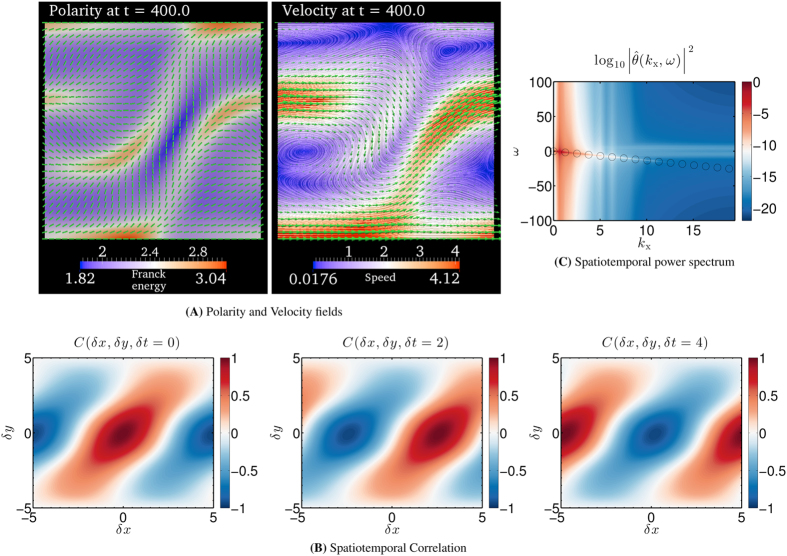
Traveling wave with traveling vortices. Spatiotemporal evolution of polarity and velocity fields for (*α*/*α*_c_) = 15. (**A**) Polarity and velocity fields after a long time *t* = 400. The polarity field is indicated by cylindrical rods. Along with the polarity field, the distortion or Franck free-energy density of the polarity field (Eq. 5) is color coded. In the velocity plot, the arrows denote local velocity normalized by the maximum magnitude of velocity across the computational domain. The direction of the arrows therefore indicate the local flow direction, and the length of the arrows indicates the relative magnitude of velocity. The one-dimensional curves are the instantaneous streamlines of the velocity field. The local speed of flow is color coded. See Sec. Model for model details and Sec. Model parameters in Sec. Methods for the parameters used to simulate the model. The horizontal direction towards the right is the positive x-direction and the vertical direction towards the top is the positive y-direction. (**B**) The color field represents the spatiotemporal correlation function 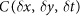) (See Sec. Spatiotemporal Correlation in Sec. Methods for the definition) for 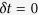, 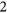 and 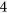. (**C**) The color field represents the logarithm of the spatiotemporal power spectrum 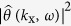 of the polarity angle *θ* as a function of angular wavenumber 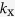 along x-direction and angular temporal frequency *ω*. 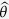 is the Fourier transform of the polarity angle *θ*. The power spectrum 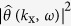 is obtained by first computing the complete normalized spatiotemporal power spectrum 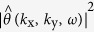, and then integrating out the dependence on the angular wavenumber 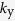 along the y-direction. The hollow circles in (**C**) trace the line 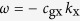 along which significant fraction of the power is concentrated.

As the activity *α* is further increased, we observe that the translational symmetry in the x-direction is spontaneously broken beyond 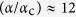. Figure 3(A) shows a snapshot of the polarity and velocity fields after a long time for 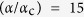 in the flow-tumbling regime. The polarity and velocity fields in Fig. 3(A) show no translational invariance in the x-direction, rendering 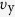 non-zero. In addition, closed streamlines of the velocity field show the presence of vortices in the flow fields.

The spatial polarity and velocity pattern observed in Fig. 3(A) travel in the x-direction with time. In order to demonstrate the traveling wave pattern, we compute the spatiotemporal correlation function 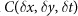 of the polarity field (see Spatiotemporal correlation in Sec. Methods). Figure 3(B) shows 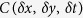 for three values of 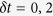 and 4. We observe that 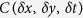 is merely translated in the x-direction as 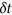 is increased beyond 0. This observation shows that the polarity field and the velocity field travel in the x-direction with time.

We next analyze the spatiotemporal frequency spectrum of the polarity field to characterize the nature of the traveling wave pattern. The spatiotemporal Fourier spectrum 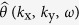 is computed as a function of angular wavenumber 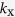 along the x-direction, angular wavenumber 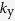 along the y-direction and angular temporal frequency *ω*. Subsequently, the power spectrum 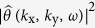 is computed normalized by the total power. Figure 3(C) shows the power spectrum 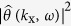 after integrating out the dependence on 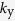. We observe that a significant fraction of the power is concentrated around 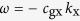 with 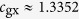. A constant 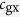 indicates that the traveling wave is non-dispersive with a constant group velocity 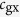 in the x-direction.

In summary, as the activity increases the contractile active fluid in the flow-tumbling regime shows oscillating spatiotemporal patterns. These spatiotemporal patterns consist of non-dispersive traveling waves accompanied by traveling vortices in the flow field. Such oscillating spatiotemporal patterns are not predicted by the linear perturbation analysis (see Critical activity *α*_c_ for spontaneous flow transition in Sec. Methods), and is therefore an effect mediated by nonlinearities in the model.

### Spatiotemporal chaos

**Figure 4:**
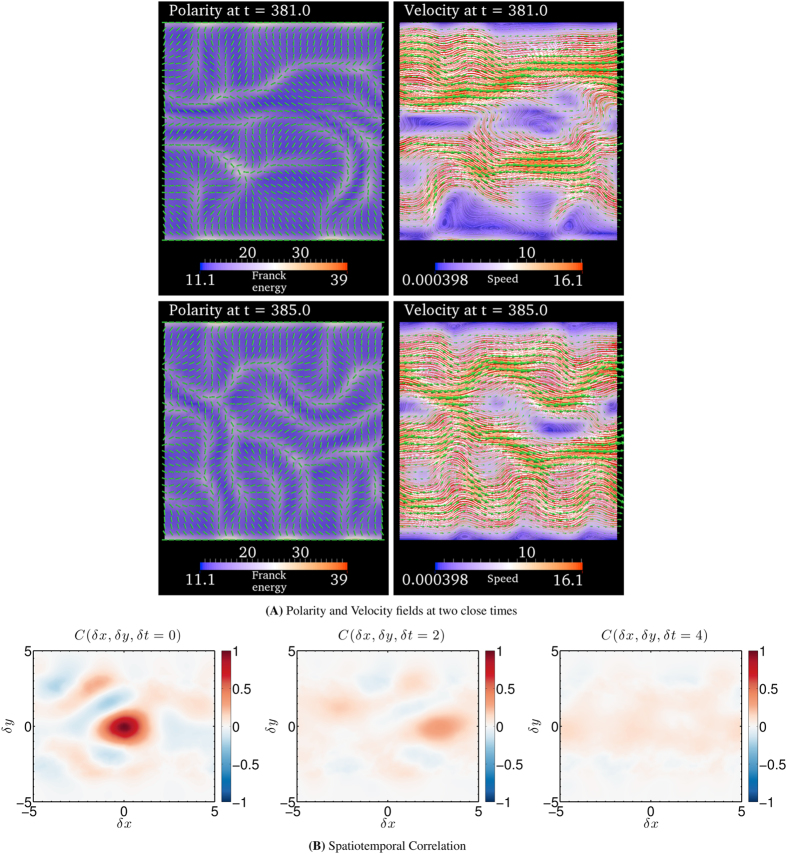
Spatiotemporal chaos. Spatiotemporal evolution of polarity and velocity fields for (*α*/*α*_c_) = 100. (**A**) Snapshot of polarity and velocity fields at times *t* = 381 and *t* = 385. The polarity field is indicated by cylindrical rods. Along with the polarity field, the distortion or Franck free-energy density of the polarity field (Eq. 5) is color coded. In the velocity plot, the arrows denote local velocity normalized by the maximum magnitude of velocity across the computational domain. The direction of the arrows therefore indicate the local flow direction, and the length of the arrows indicates the relative magnitude of velocity. The one-dimensional curves are the instantaneous streamlines of the velocity field. The local speed of flow is color coded. See Sec. Model for model details, and Sec. Parameters in Sec. methods for the parameters used to simulate the model. The horizontal direction towards the right is the positive x-direction and the vertical direction towards the top is the positive y-direction. (**B**) The color field represents the spatiotemporal correlation function 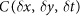 (See Sec. Spatiotemporal Correlation in Sec. Methods for the definition) for 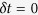, 2 and 4.

As the activity is further increased beyond 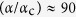, we observe that the traveling waves in the polarity and velocity fields disappear. Figure 4(A) shows the polarity and the velocity fields at two close time-points for 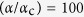 in the flow-tumbling regime. Visual inspection of the polarity and the velocity fields indicates irregular spatiotemporal dynamics. In addition, the velocity fields in Fig. 4(A) shows several vortices characteristic of a turbulent flow pattern.

In order to investigate whether the dynamics is chaotic, we compute the maximum Lyapunov exponent^32^ (see Maximum Lyapunov exponant in Sec. Methods). The maximum Lyapunov exponent *λ* is a measure of sensitivity to small perturbations, with a positive value signifying chaos^32^,^33^. We find that 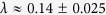. A positive maximum Lyapunov exponent indicates that a small perturbation in the polarity field gets amplified over time making the dynamics temporally decorrelated. Since the velocity field is coupled to the polarity field, we conclude that both the polarity and velocity fields shown in Fig. 4(A) are therefore temporally chaotic.

To investigate if the spatiotemporal dynamics are irregular, we compute the spatiotemporal correlation function^33^ 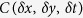 of the polarity field (see Spatiotemporal Correlation in Sec. Methods). Figure 4(B) shows 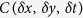 for increasing 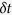. We observe that spatial correlations progressively disappear as 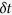 is increased. This implies that the polarity field gets spatially decorrelated in time^33^. Taken together with the positive maximum Lyapunov exponent, this shows that both the polarity and velocity fields are spatially and temporally irregular revealing characteristics of spatiotemporal chaos.

In summary, for large activities the contractile active fluid in the flow-tumbling regime exhibits spatiotemporal chaos characterized by irregular spatiotemporal patterns. Like the oscillatory dynamics, the linear perturbation analysis (see Critical activity *α*_c_ for spontaneous flow transition in Sec. Methods) does not predict spatiotemporal chaos, and is an effect of nonlinearities in the model equations.

### Comparison and summary of dynamics for flow-tumbling and flow-aligning regimes

**Figure 5:**
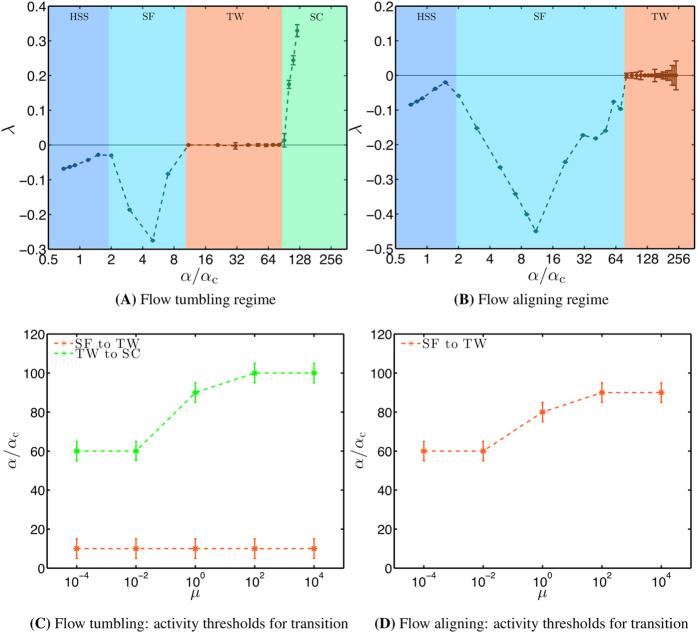
Maximum Lyapunov exponent and summary of spatiotemporal dynamics with increasing activity. (**A**) Maximum Lyapunov exponent *λ* as a function of activity 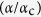 in the flow-tumbling regime. The colored regions with different labels (HSS, SF, TW, SC) characterize the spatiotemporal dynamics with increasing 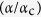. HSS: Homogeneous steady state with no flow. SF: Inhomogeneous steady state with non-zero flow. TW: Traveling wave pattern with traveling vortices. SC: Spatiotemporal chaos. (**B**) Same as (**A**) in the flow-aligning regime. Errorbars denote three times the standard deviation of the average Lyapunov exponent computed over 30,000 time windows. (**C**) As the activity 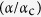 is increased, the plot shows the activity region within which transitions from steady-state with non-zero flow (SF) to traveling waves (TW), and from the TW to spatiotemporal chaos (SC) are observed in the flow tumbling case. (**D**) shows the activity region within which transitions from SF to TW are observed in the flow aligning case. In (**C**,**D**), the activity thresholds are plotted as a function of friction coefficients 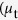, 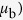 at the boundaries, assuming 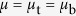.

Figure 5(A) shows the maximum Lyapunov exponent *λ* and summarizes the dynamical behavior of the contractile active polar fluid model in the flow-tumbling regime as a function of 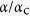. We observe that *λ* is less than 0 for 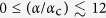 indicating that the polarity and velocity fields reach a steady state. The steady state polarity and velocity is spatially homogeneous for 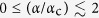 and the flow field is therefore 0 at steady state. For 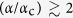, the active fluid undergoes a spontaneous flow transition. For 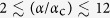, the steady state polarity field has non-zero gradients in y-direction and is translationally invariant along the x-direction. As a consequence the velocity along the x-direction is non-zero (see Sec. Spontaneous flow). For 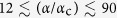, *λ* is 0 and the spatiotemporal dynamics of the active polar fluid is oscillatory. The oscillatory dynamics is characterized by traveling waves accompanied by traveling vortices in the flow field (see Sec. Traveling waves and traveling vortices). For 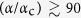, *λ* is greater than 0 indicating that the dynamics is temporally chaotic. In addition, spatiotemporal correlations disappear over a short time interval and the dynamics is therefore an instance of spatiotemporal chaos (see Sec. Spatiotemporal chaos). Thus, the contractile active polar fluid in the flow-tumbling regime undergoes 3 transitions: spontaneous flow transition, transition to oscillatory dynamics, and a transition to spatiotemporal chaos.

In contrast, in the flow-aligning regime, the contractile active polar fluid does not exhibit spatiotemporal chaos as 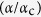 is varied over two orders of magnitude (from 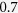 to 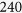). Over this range of activity the contractile active polar fluid in the flow-aligning regime only shows two transitions, namely the spontaneous flow transition and transition to oscillatory dynamics. Figure 5(B) shows the maximum Lyapunov exponent *λ* and summarizes the dynamical behavior of the contractile active polar fluid model in the flow-aligning regime as a function of 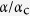. The Lyapunov exponent *λ* is less than 0 when 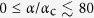. The steady state polarity and velocity fields are spatially homogeneous with no flow when 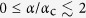. For 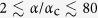, the dynamics reaches a steady state with non-zero flow in the x-direction. For 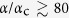, *λ* is 0 and the fluid exhibits oscillatory dynamics where the oscillations are characterized by traveling waves and traveling vortices in the flow field. Thus, the contractile active polar fluid in the flow-aligning regime undergoes 2 transitions: spontaneous flow transition and transition to oscillatory dynamics. This observation does not exclude spatiotemporal chaos at even larger activities. Nevertheless, we can conclude that range of activities showing non-chaotic dynamics in the flow-aligning regime is at least 3 times larger compared to the range of activities in the flow-tumbling regime.

Next, we investigate the effect of friction at the boundaries on the activity required for the transition from a steady-state flow to traveling waves for both the flow tumbling and flow aligning regimes. We also study the activity required for transition from traveling waves to spatiotemporal chaos for the flow tumbling regime. For simplicity, we choose the friction coefficients at both boundaries to be equal, that is 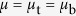. A friction coefficient *μ* = 0 corresponds a stress-free boundary while the limit of very large *μ* corresponds to a no slip boundary. Figure 5(C,D) show the activity thresholds for transition in the flow-tumbling and flow-aligning cases respectively, as *μ* is increased within a range from 10^−4^ to 10^4^. We observe that for the flow tumbling case (Fig. 5(C)), the activities required for the transition from spontaneous flow to traveling waves is independent of *μ* within the numerical uncertainty of the threshold. For the flow aligning regime (Fig. 5(D)), this threshold, however, increases before saturating at large *μ*. The activities required for the transition from traveling waves to spatiotemporal chaos that is observed only in the flow tumbling regime also increase before saturating at large *μ* (Fig. 5(C)). Nevertheless, we find that the required activities for all of the observed transitions increase only by at most 50% as *μ* is increased over 8 orders of magnitude.

## Discussion

We have studied the dynamics of active polar fluids at low Reynolds numbers as a function of a spatially-homogeneous activity measuring the strength of the active stress in the system. We consider a two-dimensional contractile, active polar fluid sandwiched between two surfaces. The surfaces impose frictional forces at the interface modeling the effect of membrane on one side and the cytosol on the other side of an actomyosin cortex in biological cells. The spatiotemporal dynamics of such an active fluid is described by a nonlinear continuum hydrodynamic description^1^,^2^,^3^. We numerically solve the hydrodynamic equations using a hybrid particle-mesh method^24^ when the fluid is subjected to activities over two orders of magnitude.

The active polar fluid not only shows transition to spontaneous flow as predicted by linear perturbation analysis, but also transitions to oscillatory spatiotemporal patterns, and even spatiotemporal chaos as the activity is increased. In the flow-aligning regime, where the polarity field tends to align with local shear, the model exhibits spontaneous flow transitions and transitions to oscillatory spatiotemporal patterns. The oscillatory spatiotemporal dynamics is accompanied by traveling waves and traveling vortices in the flow field. In the flow-tumbling regime, where the polarity field tends to tumble in local shear flow, the model also exhibits a transition to spatiotemporal chaos as the activity is increased, resulting in irregular spatiotemporal dynamics. The chaotic regime is characterized by the maximum Lyapunov exponent of the spatiotemporal dynamics which we determine numerically. The transitions are mediated by nonlinearities in the hydrodynamic description. This is the first time such transitions have been shown in active polar fluids subjected to spatially homogeneous activity. The results therefore suggest that the level of activity alone can tune the operating point of an actomyosin layer characterized by qualitatively different spatiotemporal dynamics.

These results suggest possible mechanisms for some observed biological phenomena and experimentally testable predictions. For example, oscillations and traveling waves in actomyosin cortex have been observed in amnioserosa cells during dorsal closure in *Drosophila* embryos^13^ and in periodically protruding cells^34^. Even though the presented model may be too simple for a direct comparison to these experiments, our results show that a homogeneous level of activity alone is sufficient for generating such behavior. In addition, our model predicts onset of irregular spatiotemporal dynamics as the activity is increased.

In our study, we have used several simplifications. The model is two-dimensional with the third dimension considered transitionally invariant. The finite magnitude of small perturbations to the initial condition limit the precision of numerically determining the transition thresholds. Further, we consider the case where the activity of the system is spatially homogeneous and are not regulated by other components in the system. Such regulatory mechanisms might either increase or decrease the activity needed for spatiotemporal chaos in *in vivo* actomyosin layers. We have also not considered the effect of actin filament turnover, actin polymerization and multicomponent nature of *in vivo* actomyosin layers. Intrinsic fluctuations in actomyosin layers that might be play an important role in mediating its dynamics have also not been considered^35^,^36^,^37^. Additionally, due to the constant magnitude constraint of the polarity field and the polarity boundary conditions, our model does not show topological defects in the polarity field. The effect of defects on the spatiotemporal dynamics of our model active fluid remains to be investigated. This investigation requires removing the constant magnitude constraint of the polarity field. Future work will focus on relaxing some of these simplifications to gain more insight into active polar fluids for modeling the actomyosin cortex.

We envision that a systematic investigation of such model actomyosin layers together with *in vitro* and *in vivo* experiments will help improve our understanding on the role of activity in actomyosin cortex mediating crucial morphogenetic phenomena in developing organisms.

## Methods

### Hydrodynamic equations of active polar fluids

Denoting the polarity and velocity at position 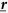 at time *t* by 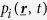 and 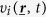 respectively 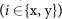, the hydrodynamic description of incompressible active polar fluids in two-dimensions assuming negligible inertial forces is made up of a constitutive relation, the Onsager relation for the polarity field, force balance condition and the incompressibility constraint^1^,^2^,^3^,^16^,^20^,^24^.

The constitutive relation of active polar fluid reads


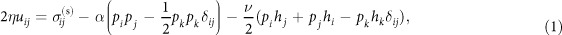


where 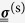 is the symmetric part of the deviatoric stress tensor with components 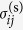, 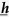 is the molecular field vector with components 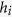, 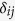 are the components of the Kronecker-delta tensor such that *δ*_*ij*_ = 1 if 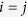 and 0 otherwise, and 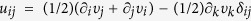 are the components of the symmetric, traceless part of the velocity-gradient tensor. In Eq. 1, the parameter 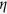 is the viscosity of the fluid, 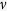 is the coefficient coupling mechanical stress to polarity field, and 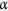 is the activity measuring the active stresses induced by consumption of energy. If *α* < 0, the active stress is extensile, and if *α* > 0 the active stresses are contractile.

The equation of motion for the polarity field is given by the Onsager relation:


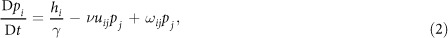


where 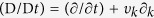 is the material (Lagrangian) derivative and 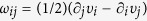 are the components of the vorticity tensor (the anti-symmetric part of the velocity-gradient tensor). In Eq. 2, the parameter *γ* is the orientational friction, and the same coefficient 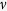 in Eq. 1 acts a coefficient between the rate of change of polarity and strain-rate 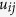. The coefficient 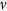 in Eq. 2 describes alignment or tumbling of the polarity field by local shear flow^2^,^17^. If 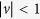, the polarity field tumbles in the local shear flow whereas for 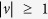 the polarity field tends to align with the local shear flow.

The force balance condition and the incompressibility constraint are given by


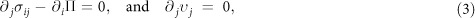


respectively. Here, 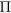 is the pressure and 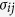 is one of the four components of the deviatoric stress tensor 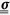. The deviatoric stress 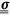 is a sum of a symmetric stress 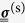 (Eq. 1), an antisymmetric stress 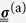:


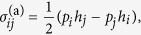


and the Ericksen stress 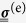 which is an equilibrium stress generalizing the hydrostatic pressure to anisotropic fluids^38^,^39^.

The components 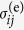 of the Ericksen stress tensor and the components 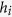 of the molecular field vector 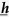 are defined as a function of a distortion free-energy density *f*:


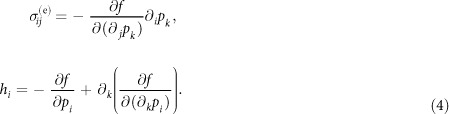


The distortion or the Franck free-energy density defines the increase in the energy density due to distortions in the polar nematic liquid crystals from its uniformly aligned configuration and is defined as


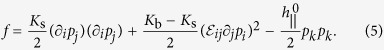


where 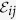 are the components of the permutation (Levi-Civita) tensor. The free-energy density 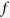 is parametrised by 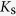, the splay elastic constant and 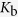, the bend elastic constant. We neglect the twist elastic constant since it is irrelevant in two dimensions. The free-energy density also includes a contribution from a component 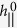 of the molecular field assuming that fluctuations in polarity orientation dominate the fluctuations in polarity amplitudes. This assumption implies that the amplitude 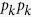 is a constant and can be assumed to be 1 without loss of generality^2^. Using Eqs 4, 5 and ensuring 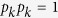,


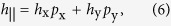


where 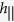 is the parallel component of the molecular field that includes contribution from 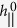 in Eq. 5. The transverse component of 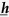, 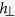, creates a torque that tends to align the polarization field. It is given by


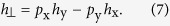


Using Eqs 6 and 7,


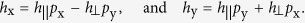


Substituting these expression for 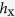 and 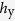 in Eq. 2 and setting 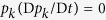 to ensure that 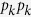 stays constant, we find that


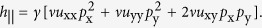


Equations 1, 2 and 3, along with boundary conditions and initial polarity field fully describe the hydrodynamics of incompressible active polar fluids. We numerically solve these equations using a recently developed hybrid particle-mesh method for incompressible active polar fluids. For details on the computational method, refer to Ramaswamy *et al.* (2015)^24^.

### Critical activity *α*_c_ for spontaneous flow transition

We consider an active polar fluid that is translationally invariant along the x-direction and has a thickness 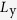 in the y-direction. The surface of the fluid at 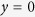 and 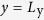 is impenetrable (
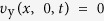
 and 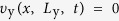), and has a friction boundary condition so that 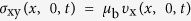 and 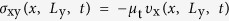 where 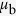 and 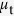 are the friction coefficients. The polarity at *y* = 0 and 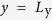 is parallel to the x-axis so that 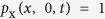, 
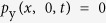
 and 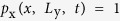, 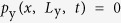, so that the polarization angle 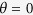 along 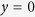 and 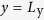. In addition, we assume that the elastic constants 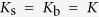.

The incompressibility constraint, along with translational invariance in the x-direction and impenetrable surfaces at *y* = 0 and 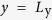 render 
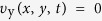
. The translational invariance along the x-axis and the force balance condition render 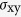 constant along the y-axis. Evaluating the hydrodynamic equations at steady-state, the polarization angle 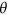 (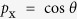 and 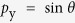) and the only non-zero velocity gradient 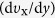 are given by


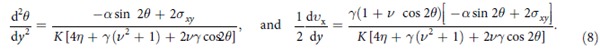


A trivial solution to these equations (satisfying the boundary conditions) at steady state is 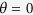 (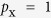 and 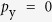) and 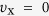. Expanding Eq. 8 around a small perturbation *ε* to the steady state configuration of polarity, and using the polarity and friction boundary conditions at 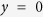 and 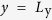, we find that *ε* is finite and non-zero for activities 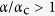 where the critical activity 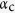 is within an interval given by


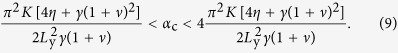


For activities 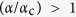, the polarity *θ* is finite leading to a spontaneous flow transition since 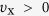 due to gradients in polarity along the y-direction^2^,^18^,^22^,^24^. This transition is similar to the classical Fréedericksz transition of nematic liquid crystals in which the transition is brought about by an external magnetic field and not by internal active stresses^2^,^18^,^38^.

### Model parameters

We define the parameters in our model scaled by units for length *l*, time *τ* and stress *σ*. The time unit 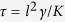 where the elastic constants 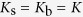 in Eq. 5. The unit for stress 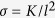. The unit for length 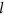 is chosen to be 1. In these units, we choose viscosity 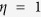. The dimensionless coupling coefficient 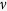 is chosen in order to reflect properties of contractile^11^,^14^ and spontaneously flowing^7^,^8^,^9^ active systems like actomyosin gels. In our hydrodynamic description (Sec. Hydrodynamic equations of active polar fluids), contractile active stress in Eq. 1 requires that activity 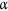 contributes to positive stress in the direction of polarity^2^,^18^. Positive active stress parallel to polar direction is ensured when 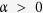. In addition, for contractile active fluids to exhibit spontaneous flow for 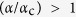, the critical activity 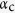 (Eq. 9) needs to be positive. Positive critical activity is ensured if 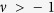 in Eq. 9. Therefore, contractile active polar fluids capable of exhibiting spontaneous flow can be realized in a flow-tumbling regime where 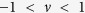 or in a flow-aligning regime where 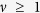
2,17,18,22. For the flow-tumbling regime, we choose 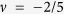 and for the flow-aligning regime, we choose 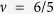.

#### Friction at the boundaries

We impose a friction boundary condition along 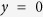 and 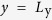 such that the shear stress 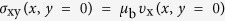 and 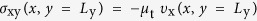. We set 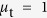. Assuming that the friction coefficients are different at the two surfaces in general, we set 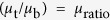. Without loss of generality, we assume that the friction coefficient 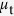 at 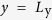 is greater than or equal to the friction coefficient 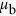 at 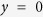. Therefore, 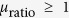. For all simulations presented in the paper, we set 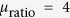. We, however, verified that the results are qualitatively unaltered as 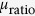 is varied between 1 to 10.

### Settings for numerical simulation

For the numerical simulation using the hybrid particle-mesh method^24^, we use 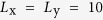, and discretize the computational domain into 65 mesh nodes in each direction. The time integration is performed using a time-step length of 0.0004 using fourth-order Runge-Kutta time integration scheme^24^.

The initial condition for the polarity field 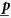 is a trivial spatially homogeneous steady state of the model active polar viscous layer except for a small perturbation to the polarity field at the centre of the computational domain. The trivial steady state of the hydrodynamic model under the boundary conditions used for the model active polar viscous layer is 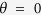 (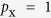 and 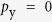) over the entire spatial domain. This trivial steady state is perturbed by 1% at the centre by setting 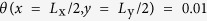 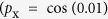 and 
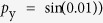
. The resulting polarity field is used as the initial condition in all the simulation presented in the paper.

### Spatiotemporal correlation

We define the spatiotemporal correlation function 
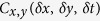
 of the polarity field 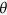 as:


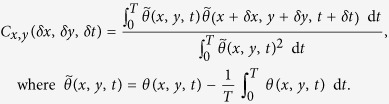


The spatiotemporal correlation function 
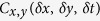
 is centred at an arbitrary point 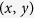 in the computational domain, and 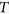 is the time window over which the correlation is computed. The range of 
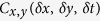
 is between −1 and 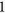. A value of −1 indicates perfect negative correlation, a value of 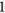 indicates perfect positive correlation and a value of 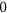 indicates perfect decorrelation. In Sec. Results, we report the spatiotemporal correlation function 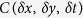 with respect to the centre of the computational domain at 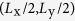. That is, 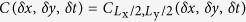.

### Maximum Lyapunov exponent

The maximum Lyapunov exponent of the spatiotemporal dynamics of the active polar fluid model is computed using Benettin’s standard method^32^,^40^. The Lyapunov exponent is computed over a time interval of 0.02 consisting of 50 numerical time-integration steps. The computation of the Lyapunov exponent is performed as a function of time until the Lyapunov exponents from the final 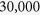 time intervals are samples from a stationary distribution.

## Additional Information

**How to cite this article**: Ramaswamy, R. and Jülicher, F. Activity induces traveling waves, vortices and spatiotemporal chaos in a model actomyosin layer. *Sci. Rep.* **6**, 20838; doi: 10.1038/srep20838 (2016).
